# A socioeconomic disadvantage index for informing policy, systems, and environmental change interventions for senior nutrition programs

**DOI:** 10.3389/fpubh.2025.1520925

**Published:** 2025-01-21

**Authors:** Shadi Maleki, Cassandra M. Johnson, Francis A. Méndez Mediavilla, Ramalingam Shanmugam, Lesli Biediger-Friedman

**Affiliations:** ^1^Translational Health Research Center, Texas State University, San Marcos, TX, United States; ^2^Nutrition and Foods Program, School of Family and Consumer Sciences, Texas State University, San Marcos, TX, United States; ^3^Department of Information Systems and Analytics, Texas State University, San Marcos, TX, United States; ^4^School of Health Administration, Texas State University, San Marcos, TX, United States

**Keywords:** GIS, food insecurity, risk factors, basic needs, descriptive study

## Abstract

Identifying socioeconomic disparities within a local area is critical for tailoring policy solutions to older adult populations. However, a comprehensive index for characterizing socioeconomic disadvantage for older adult households in the U.S. is yet to be developed. This study is the first of its kind and used secondary data from the U.S. Census to develop a new socioeconomic disadvantage index for adults older than 60 years, with a focus on food insecurity, for a large region in Central Texas. The Older Adult Socioeconomic Disadvantage Index (OASDI) includes 12 variables related to unmet needs for food, housing, healthcare access, and transportation, and others at the census tract level. For each variable, the values were ranked based on quintiles using ArcGIS Pro 3.2. An unweighted sum was used to create the OASDI, where a higher score indicated greater socioeconomic disadvantage. Choropleth maps were used to visualize the OASDI and persistent poverty for all census tracts within the study area. The OASDI was used to statistically compare two local policy regions for senior nutrition programs in the Austin and San Antonio, Texas metro areas. Results showed a greater socioeconomic disadvantage in the San Antonio region compared to the Austin region (Mann-Whitney-U = 198,303; *p* < 0.0001). The statistical analysis identified an area with extreme disadvantage relative to the local policy region and confirmed with member checking. Findings provided insights into local socioeconomic disparities at different levels and can be applied to advocate for policies, systems, and environmental changes for senior nutrition.

## Introduction

The United States (U.S.), like other countries, has a large and growing population of older adults, individuals aged ≥60 years and older. Based on projections from the U.S. Census Bureau (Census), the proportion of older adults is expected to outnumber children in 2034 (1). By 2060, the older adult population may represent one quarter (25%) of the U.S. population (2).

Food insecurity is an important health determinant for older adults (3). The U.S. Department of Agriculture (USDA) defines food insecurity as a household-level economic and social condition, characterized by “limited or uncertain availability of nutritionally adequate and safe foods, or limited or uncertain ability to acquire acceptable foods in socially acceptable ways” (4). The USDA annual food insecurity report stated that 13.5% of all U.S. households experienced food insecurity at some point in 2023, which is higher compared to 2022 data (5).

Previous studies have reported on a relatively high burden of food insecurity for older adults, particularly for those living in low-income households or communities (3, 6–8). Data from the Current Population Survey revealed that 5.5 million older adults (above 60 years old) were food insecure in 2021 (9), and estimates of food insecurity may be higher than estimates due to survey and sampling limitations (10).

Food insecurity among older adults is a complex issue influenced by social (e.g., living alone), physical (e.g., physical or functional limitations), and systemic factors (e.g., access to food or health care services) (11, 12). Older adults are more susceptible to poverty and may be unable to meet basic needs for health and wellbeing (13). Poverty affects all dimensions of wellbeing and hampers access to basic needs, including food (14). Other factors, including gender (15), race and ethnicity (16), disability (17), living alone (10), having limited access to transportation (18) and health insurance (19), and challenges with housing (20), are associated with food insecurity among older adults. In addition, scholars have established associations of food insecurity with adverse outcomes, including malnutrition (21, 22), and advocated for strengthening food and nutrition programs that address food insecurity among older adults (6, 23).

Public health policies and programs must be evidence-based, equity-oriented, and designed with implementation in mind (24). However, there is limited local data on food insecurity available for researchers to understand food insecurity within their communities or understand food insecurity as one of several unmet basic needs important to health and wellbeing. Previous studies have outlined key considerations (13) or developed measurement tools for assessing food insecurity among older adults, including an expanded food security screener (10, 25, 26). Recently, Lee et al. created the Older Adult Food Insecurity Index to assess food insecurity within a geographic area (10). Their study built an index with 13 risk factors of food insecurity based on prior studies and guided by the Socioecological Model and they tested the index for a county in Hawaii. While their index focused on risk factors for food insecurity, it did not include other indicators of socioeconomic disadvantage, such as unmet transportation needs, which are important for understanding food insecurity among older adults, especially in a large state such as Texas.

In 2022, the Administration for Community Living (ACL) funded an interdisciplinary research project, called Nutrition for Underserved Elders via Application or NUEVA, to develop, implement, and evaluate a new multi-function app for innovating the existing senior nutrition programs in Central Texas (https://www.fcs.txst.edu/nutrition/nueva.html). The state of Texas has a growing number of older adults. According to 2023 Profile of Older Americans, there was a 41.8% increase from 2012 to 2022 in Texas's older adult population, and 12% percent were below poverty in 2022 (27). Texas is one of the states with the highest concentration of adults 65 years and older (27). Texas was one of the seven states where food insecurity was much higher compared to the national average (16.9% statewide vs. 12.2% nationwide prevalence over a 3-year period 2021–2023) (5). The NUEVA project is situated in San Marcos, Texas, a city located between two of the largest cities in the U.S.—Austin and San Antonio and near small towns and rural communities. Two local organizations, the Alamo Area and Capital Area Councils of Government (COG), and their respective Area Agencies on Aging (AAA) coordinate senior nutrition services for older adults living in Central Texas.

Inspired by Lee et al. (10), this study developed a comprehensive index for socioeconomic disadvantage—the Older Adult Socioeconomic Disadvantage Index (OASDI)—to understand risk for food insecurity among older adults living in Central Texas. The aim of this article is to describe the creation of a new index for socioeconomic disadvantage for households with older adults that considers unmet basic needs for food, housing, medical/healthcare, and transportation, in addition to well-established risk factors of food insecurity or nutritional risk for older adults, such as experiencing disabilities or living alone. The primary aim is to use this new index to identify disparities within and across a large geographic region. Findings from this study can inform coordinated efforts for creating new programs or strengthening funding or outreach for existing programs and motivating future capacity-building research for older adults or seniors.

## Methods

### Setting

**Figure 1:**
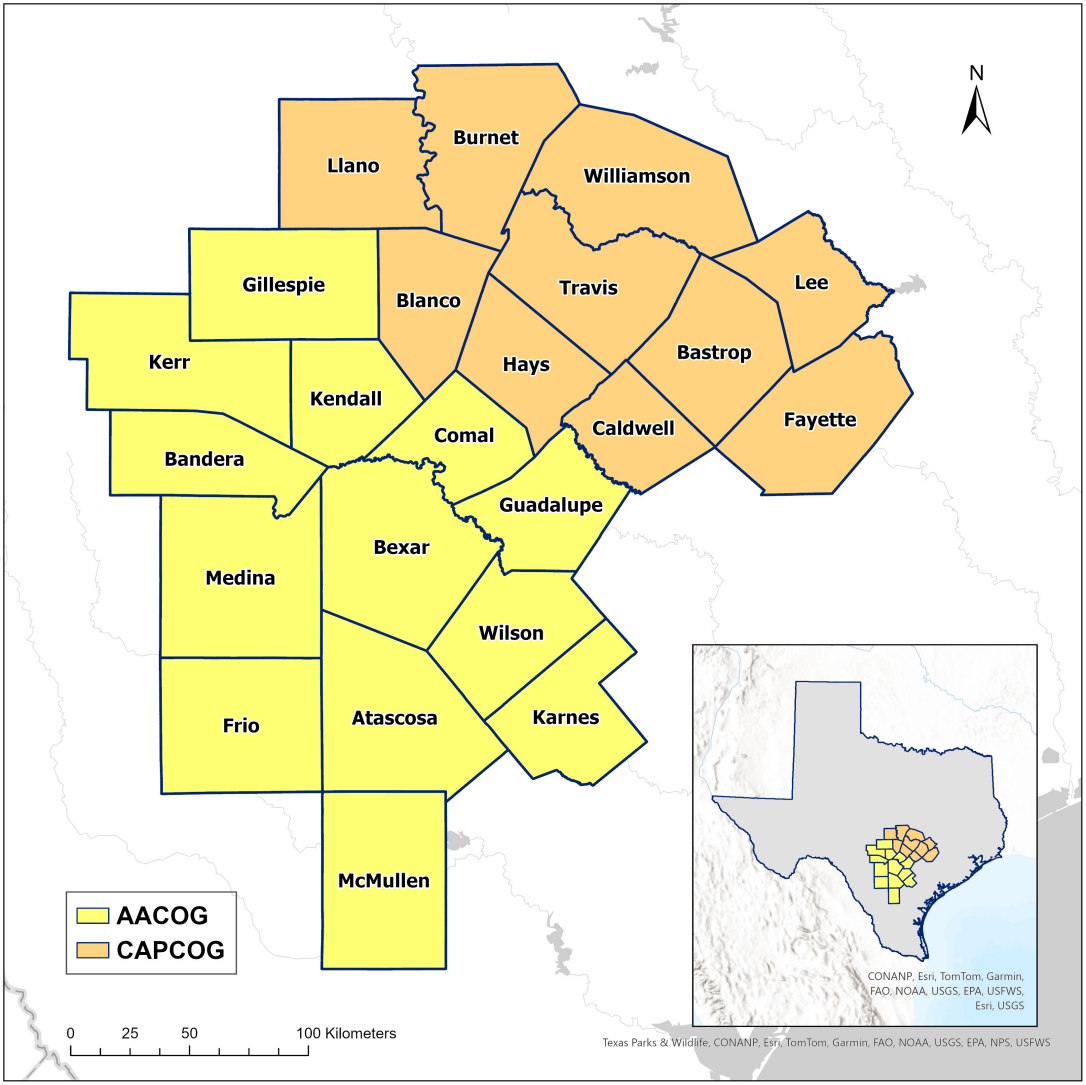
Study area for the NUEVA Project in Central Texas. The larger map shows the 23 individual counties in Central Texas and the two planning regions of CAPCOG and AACOG. The smaller map, located in the bottom right corner, shows the study area within the state of Texas. CAPCOG, Capital Area Council of Government; AACOG, Alamo Area Council of Government.

The study area consists of 23 rural and urban counties comprised of 1,077 census tracts (CTs). The 23-county area is defined based on the two local policy and administrative regions, relevant for senior nutrition, for the Austin and San Antonio, Texas metropolitan (metro) areas. The AAA of the Capital Area and Capital Area Council of Government (CAPCOG) serves 10 counties, including those in the Austin Metro Area, and Bexar Area AAA and Alamo Area Council of Government (AACOG) serves 13 counties, including the San Antonio Metro Area (Figure 1). Austin was one of the fastest-growing large metro areas in the U.S. for 12 consecutive years until 2022, and the population of Austin-Round Rock-San Marcos Metropolitan Statistical Area (MSA) increased by 50,000 residents between 2022 and 2023 at a rate of 2.1 % (28). In 2023, the city of San Antonio ranked the third-fastest growing city in terms of population (29), and the Austin-San Antonio metro population is projected to increase by over 3 million by 2050 (30).

### Index development

An interdisciplinary team of investigators with expertise in geography, statistics, and public health developed an index, called the Older Adult Socioeconomic Disadvantage Index (OASDI), using an a priori approach. Drawing from prior methodological approaches by Kaczynski et al. (31), and related literature on food insecurity among older adults (3, 8, 11, 32), including a previous index by Lee et al. (10), the authors constructed the OASDI index using 12 variables based on a review of the existing literature on senior nutrition and food insecurity and consultation with nutrition experts. By design, the index includes variables for the different domains of material hardship, such as food, housing, health/medical care, and transportation, and well-established indicators of socioeconomic disadvantage for older adults, such as identities based on race or ethnicity, gender, educational attainment, social isolation, disabilities (or disability status), and poverty. Material hardship is defined as unmet basic needs for food, housing, medical/health care, and transportation (33). Previous studies have established this set of socioeconomic characteristics as risk factors for food insecurity and nutritional risk among older adults (16).

The Census provided data for index creation, which were downloaded from the *Simply Analytics* (https://simplyanalytics.com/) database accessed through the university's library. All variables were based on 2021 data. Table 1 describes each variable of interest and the data source. There were two variables where proxies were used because of limitations with Census data. First, unmet need for food was operationalized as households with adults, 60 years or older, participating in the Supplemental Nutrition Assistance Program (SNAP). SNAP is the largest and most important federal food assistance program in the U.S. (5). Households participating in SNAP tend to experience more financial precarity than income-eligible households that do not participate in the program (34). A previous study reported that older adults participating in SNAP were more food insecure compared to their counterparts (35). For the index, this proxy variable—households with an adult aged ≥60 years participating in SNAP—is an indicator of unmet need for food among senior households. Second, the Census does not collect data to assess unmet need for housing, such as the proportion of disposable income paid in monthly rent or mortgage, or substandard housing conditions (36), even though renting is associated with financial precarity compared to owning among older adults (37). Other studies have used the proportion of renter-occupied households as a marker of socioeconomic disadvantage (7, 38–40). For the index, unmet need for housing was operationalized as households with adults >65 years that are renter occupied. In both cases, the food and housing variables selected for the index are the most relevant variables in the Census data.

**Table 1:** List of conceptual and operational variables in the older adult socioeconomic disadvantage index (OASDI) and data sources.

**Conceptual variable**	**Operational variable**	**Data source**
Gender	Female ≥65 years	Census [B01001]
Race or ethnicity	Black ≥65 years	Census [B01001B]
	Hispanic or Latino ≥65 years	Census [B01001I]
	Seniors ≥65 years with medicare only	Census [B27010]
Education	Less than high school graduate ≥65 years	Census [B27019]
Disability status	Seniors ≥65 years with disability	Census [B18101]
Social isolation	Seniors ≥60 years living alone	Census [B09021]
Food	Seniors ≥60 years receiving food assistance with supplemental nutrition assistance program	Census [B22001]
Housing	Renter occupied >65 years	Census [B25015]
Medical/health care	Seniors ≥65 years without health insurance	Census [B27001]
	Seniors ≥65 years with medicare only	Census [B27010]
Transportation	Seniors ≥60 years with no vehicle	Census [B25045]
Poverty status	Seniors ≥60 years in poverty	Census [B17001]

The 12 index variables covered indicators of socioeconomic disadvantage based on gender, race or ethnicity, education, and disability status; domains of material hardship, defined as unmet basic needs for food, housing, medical/health care, and transportation; and poverty. Data for each variable came from U.S. Census Bureau (Census), which are available online. Different variables used a different age number to define older adult (e.g., based on 60 years or 65 years). The number in brackets is the Census data table number.

This study calculated the OASDI at the census tract level for the 23 counties in the study area in Central Texas. As stated previously, the 23-county study area represents the study area for a grant-funded project to innovate senior nutrition programs. A multi-level analysis provides necessary context for understanding socioeconomic disadvantage of the Central Texas region within the state. For each variable, the values for all census tracts in Texas were ranked based on a quintile classification method in ArcGIS Pro 3.2. Thus, each tract received a rank between 1 and 5, where 1 means that the variable's value for that tract falls within the first quintile (least disadvantaged) and 5 refers to the fifth quintile (most disadvantaged). Ultimately, the mean value of the sum of the ranks was calculated for each census tract to represent the index value for each tract. All the variables were weighted equally and framed negatively to show greater disadvantage. A higher index score indicates greater socioeconomic disadvantage. Choropleth maps were used to visualize the index at the census tract level across 23 counties. The same process was used to reproduce the index at the county level across the 23 counties of the study area. Each county was ranked based on the quintile classification method and the mean of the sum of the ranks for the 12 variables was calculated to represent the index value for each county.

To facilitate interpretation for the OASDI, persistent poverty data were added to the index map. Persistent poverty marks census tracts and counties with a history of high poverty, defined as ≥20%, over approximately the past 30 years (41). The U.S. Census provided data on persistent poverty at the census tract and county levels between 1989 and 2019 (41). Due to the mismatch between the geometry files from 2019 and 2021, the authors were not able to visualize the one census tract in Frio County affected by persistent poverty. For this study, the authors mapped the distribution of the older adult population at the census tract level with an overlay of persistent poverty. Mapping distribution of the older adult population and persistent poverty provided critical information for interpreting the OASDI. In addition, the authors used member checking with the NUEVA project team and community partners to help interpret the distribution of the index scores.

Development of the index and visualization of its distribution was conducted in ArcGIS Pro 3.2 product of Environmental Systems Research Institute (Esri) headquartered in Redlands, California (Available online: https://www.esri.com/en-us/arcgis/geospatial-platform/overview). The software has been accessed through the university's license.

### Statistical analysis and testing

**Figure 2:**
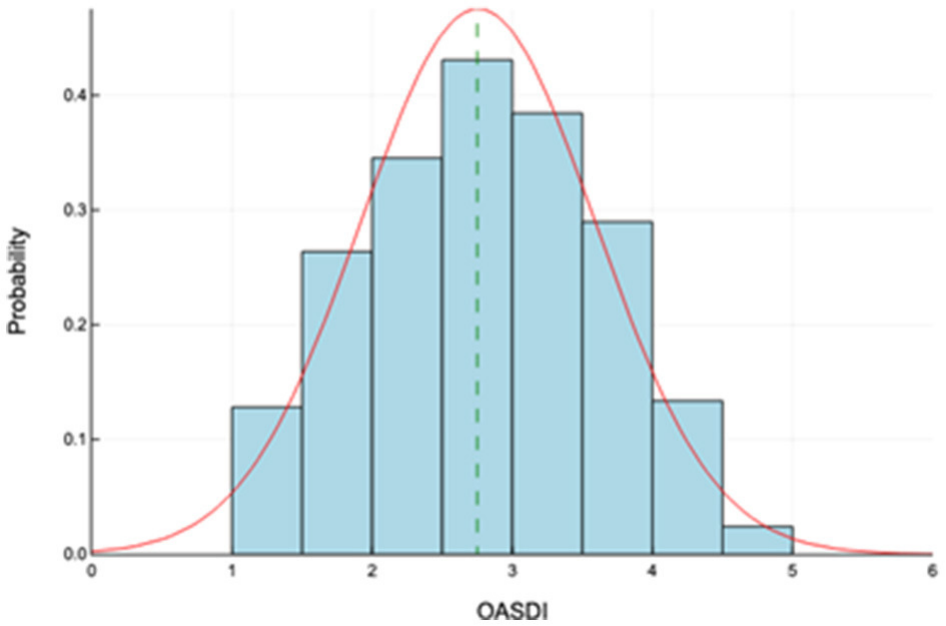
Distribution of the older adult socioeconomic disadvantage index (OASDI). This figure shows the probability distributions of OASDI for the 23 counties under study (range: 1 to 5); the vertical dashed lines in green show the median OASDIs.

The authors hypothesized that there would be a difference in socioeconomic disadvantage between the counties that comprise the two policy regions: AACOG and CAPCOG (Figure 1). If this is the case, that difference should be captured by comparing the median index scores for these regions. Figure 2 shows the distributions of OASDI for each planning region, AACOG and CAPCOG. The distributions of OASDI seem to suggest a difference in socioeconomic disadvantage between these two policy regions. A test of significance was used to assess the difference in socioeconomic disadvantage between AACOG and CAPCOG (31). A non-parametric Mann-Whitney U test was used to assess whether there is a difference between the median index scores for these two metro policy areas. Data manipulation and statistical analyses were completed using Julia language, an open-source statistical software for data science (42).

## Results

Descriptive statistics for the Older Adult Socioeconomic Disadvantage Index (OASDI) are shown in Table 2. The analysis revealed no outlier tracts within the 23-county study area. OASDI scores seem normally distributed (Shapiro-Wilk Test *p* = 0.25, *n* = 100) with a mean score of 2.76 and some tracts scoring relatively close to the maximum (i.e., 4.92 vs. 5) for the state of Texas. Figure 2 displays the distributions of the OASDI by census tract within the 23 counties of the study area. Farther from these metro areas, in more rural counties, scores increase, meaning greater socioeconomic disadvantage among older adults. The maps also show census tracts that experienced persistent poverty between 1989 and 2019. In the Austin metropolitan area, socioeconomic disadvantage is more pronounced in the eastern tracts, known as “East Austin” or the “Eastside,” as well as the Del Valle area and other neighborhoods. In the San Antonio metro area, the most socioeconomically disadvantaged tracts are more concentrated in the central part of Bexar County. For some census tracts, the persistent poverty designation aligns with higher OASDI scores, indicating relatively higher socioeconomic disadvantage among households with older adults. However, there are also census tracts with lower OASDI scores, and thus lower levels of socioeconomic disadvantage among older adults, that are identified as persistent poverty areas. These tracts are in Hays County, west of Austin in Travis County, and the outer areas of San Antonio in Bexar County.

**Table 2:** Distribution of the older adult socioeconomic disadvantage index (OASDI) by policy region.

**Region**	**Mean**	**Median**	**Range**	**Standard deviation**
AACOG	3.0	3.1	[1, 4.9]	0.77
CAPCOG	2.5	2.4	[1.0, 4.8]	0.82

This table presents descriptive statistics for the distribution such as mean, median, range, and standard deviation by region for the 23-county region. The AACOG includes the San Antonio metropolitan area. The CAPCOG region includes the Austin metropolitan area. AACOG, Alamo Area Council of Government; CAPCOG, Capital Area Council of Government.

**Figure 3:**
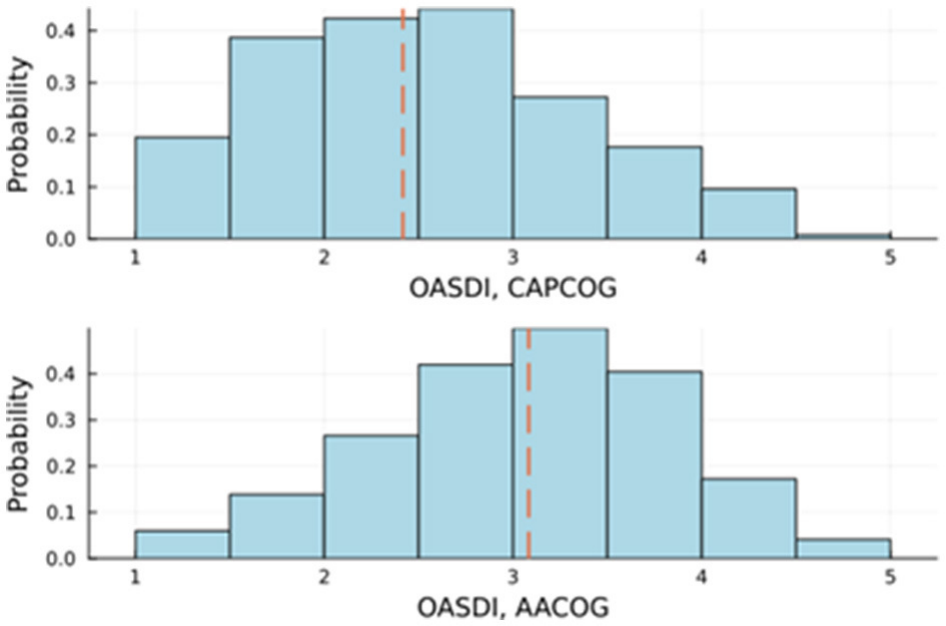
Comparison of the older adult socioeconomic disadvantage index (OASDI) across two regions in Central Texas. This figure shows the probability distributions of OASDI for each planning region (range: 1 to 5); the dashed lines show the median OASDIs. The AACOG includes the San Antonio metropolitan area. The CAPCOG includes the Austin metropolitan area. AACOG, Alamo Area Council of Government; CAPCOG, Capital Area Council of Government.

**Figure 4:**
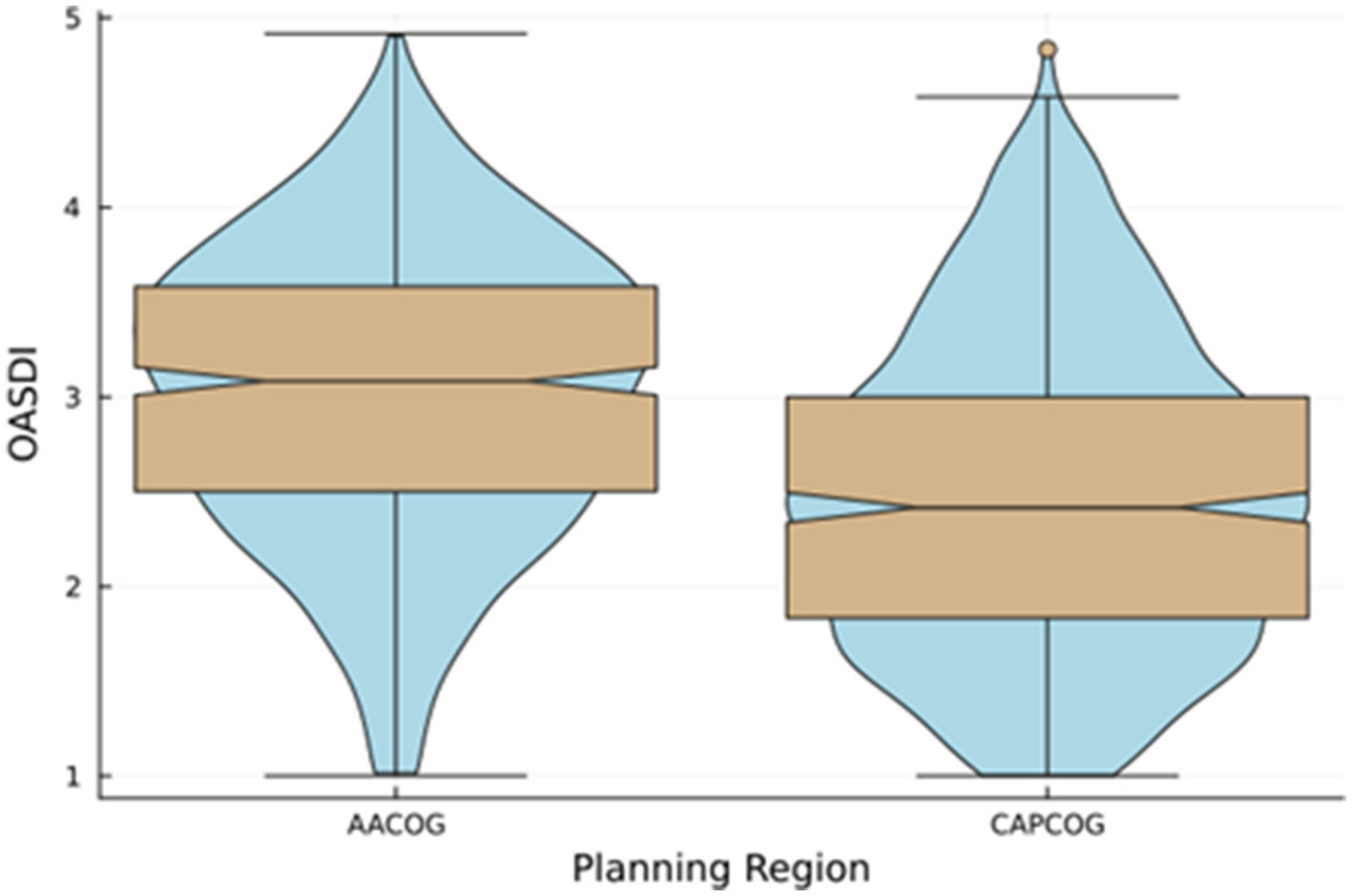
Comparison of the older adult socioeconomic disadvantage index (OASDI) across two regions in Central Texas. This figure shows a violin plot with a boxplot overlay and a comparison of the density of OASDI for the policy regions. The dent represents the confidence interval. The horizontal lines at the top and bottom represent the minimum and maximum values of the OADSI score. For the CAPCOG region, the gold circle represents an outlier. The AACOG includes the San Antonio metropolitan area. The CAPCOG includes the Austin metropolitan area. AACOG, Alamo Area Council of Government; CAPCOG, Capital Area Council of Government.

Figure 3 also shows the visual comparison of the distributions of the OASDI across the two policy regions. The OASDI distribution for CAPCOG is somewhat positively skewed. For CAPCOG, the interquartile range is shifted toward the lower values of the OASDI. A Mann-Whitney U test of significance showed a statistically significant difference between the median scores of AACOG and CAPCOG. The findings suggest that there is a difference in social disadvantage for these two policy regions. Overall, a greater socioeconomic disadvantage is detected in the San Antonio region (AACOG) compared to the Austin region (CAPCOG) (Mann-Whitney U = 198,303; *p* < 0.0001). However, the positive skewness of the distribution of the OASDI for CAPCOG denotes an area of relative extreme disadvantage within CAPCOG. Indeed, Figure 3 shows an outlier OASDI for a census tract corresponding to CT 48209010401, which is in Hays County. This tract has a significantly higher OASDI score compared to the rest of the tracts in CAPCOG area; however this tract has not been identified as an area in persistent poverty. Figure 4 shows violin plots with corresponding overlays of boxplots. The violin plots show the differences in skewness of the OASDI for each policy region, whereas the notches in the boxplots display the statistical difference in the median OASDI for the corresponding policy regions. It can be visualized that the median value of the OASDI score is higher for AACOG compared to CAPCOG. The confidence intervals do not overlap, and an outlier exists within the CAPCOG policy region. There were no outliers within the AACOG region (Figure 4). It was found that, overall, lower ranking census tracts, that indicates less socioeconomic disadvantaged tracts, are clustered around the Interstate 35 corridor between San Antonio and Austin metro areas (Figure 4).

**Figure 5:**
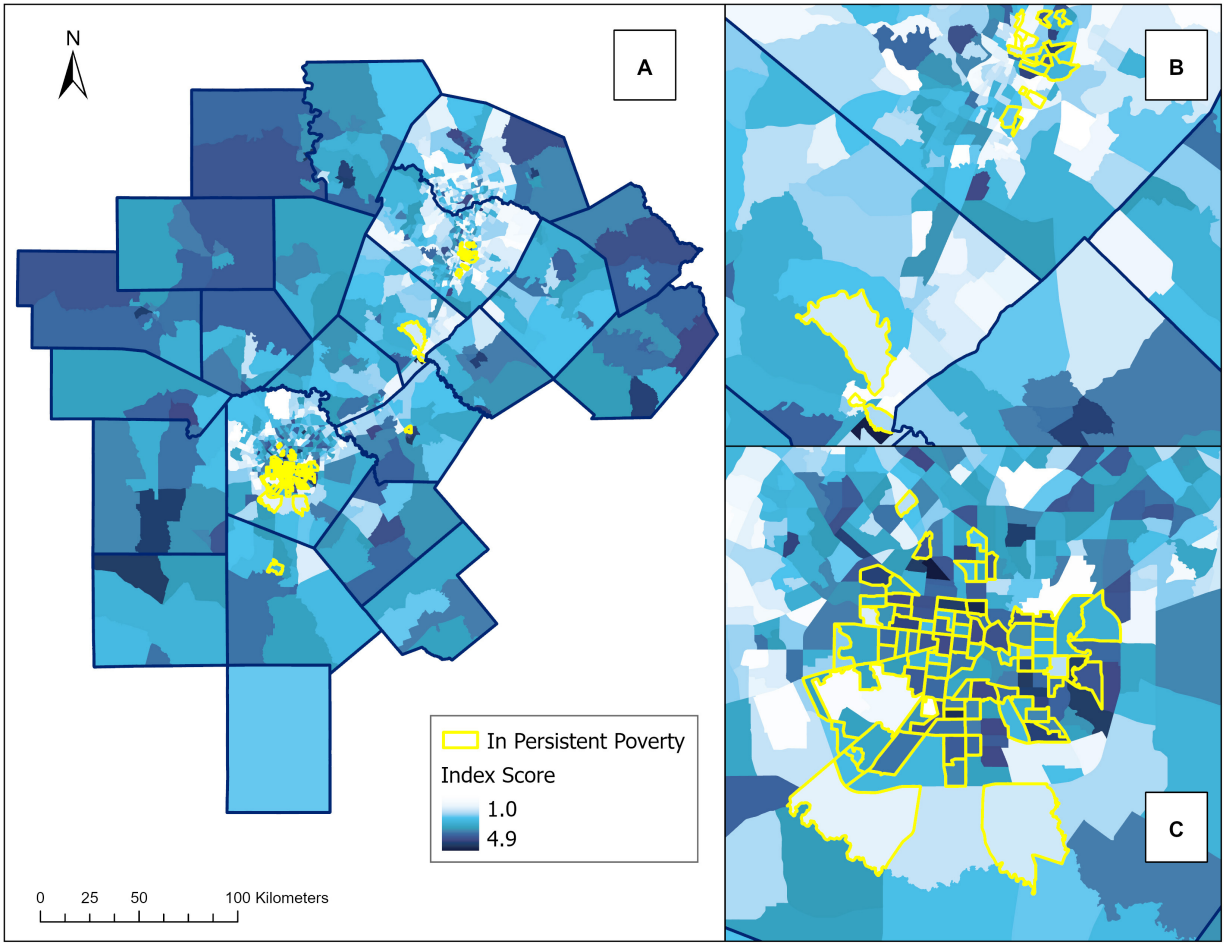
Spatial distribution of the older adult socioeconomic disadvantage index (OASDI) index scores and persistent poverty by census tract. This figure shows the 23-county study area in Central Texas. Persistent poverty was not available for all census tracts in all counties. For example, Frio County had missing data on persistent poverty at the census tract level. Choropleth maps were used to represent the index score which is calculated as the mean of the summed quantile ranks of the 12 selected variables for each census tract. Darker colors indicate a higher index score, representing greater socioeconomic disadvantage, while lighter colors indicate a lower index score, signifying lower socioeconomic disadvantage. The highlighted census tracts indicate areas of persistent poverty. **(A)** Of the map shows the index distribution across the 23 counties in the study area. **(B)** Shows a close-up view of the index distribution in Austin, Travis County. **(C)** Shows a close-up view of the index distribution in San Antonio, Bexar County.

**Figure 6:**
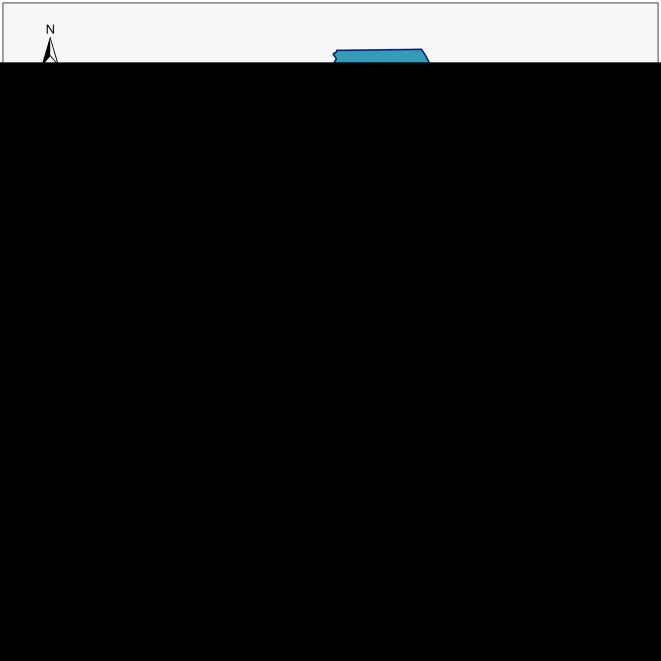
Spatial distribution of the older adult socioeconomic disadvantage index (OASDI) index scores and persistent poverty by county. This figure shows the 23-county study area in Central Texas. Choropleth maps were used to represent the index score which is calculated as the mean of the summed quantile ranks of the 12 selected variables for each county. Darker colors indicate a higher index score meaning greater socioeconomic disadvantage; the lighter colors indicate a lower index score meaning lower socioeconomic disadvantage. The yellow hatching indicates persistent poverty at the county level.

The index and persistent poverty data are also mapped at the census tract (Figure 5) and county level (Figure 6). These maps offers a high-level overview of the patterns. Specifically, at the census tract level, the index distribution indicates lower overall socioeconomic disadvantage in Austin metropolitan area compared to the San Antonio metropolitan area. Of the 23 counties in the study area, only one county, Frio, was designated as a county with persistent poverty (Figure 6). This classification is due to the Census' criterion, which designates a county as having persistent poverty if it has maintained poverty rates of 20 % or more for the past 30 years.

## Discussion

This study developed a new index, the OASDI, to assess socioeconomic disadvantage among adults aged 60 and older in Central Texas. The index is composed of socioeconomic and demographic variables that have been shown to impact food security in older adults. Thus, the index helps identify census tracts and counties where older adults are more likely to experience food insecurity, social isolation, or other adverse health outcomes, due to socioeconomic disadvantage. Findings revealed significant disparities at multiple levels, which emphasized the need for collaborative efforts at the community, state, regional, and federal levels to address food insecurity among older adults. In the Austin metropolitan area, patterns of disadvantage align with the city's overall sociodemographic profile and its history of gentrification (43). While Westside San Antonio has historically been affected by generational poverty (44), this study's findings reveal greater levels of disadvantage in other neighborhoods like the South and East Sides of San Antonio, as well as the West Side. The pattern of greater socioeconomic disadvantage in areas more distant from Interstate-35 suggests that specific attention is warranted to address transportation challenges, which can significantly impact lower-income senior residents, consistent with a previous recent study (9).

Like another study (10), this study considered the literature, integrated theory, and utilized publicly available secondary data to create a replicable index based on multiple factors. Lee et al. applied the Socioecological Model to develop the Older Adult Food Insecurity Index, but their index did not include a variable for transportation, though they included a variable for percentage of the senior population living at least half a mile from a grocery store (10). There were additional differences in their operationalization of variables related to food and housing and their use of weights. Lee et al. derived weights from a literature review, while this study weighed all factors equally. They applied the index at the county, zip code, and census tract levels for Honolulu County in Hawaii (10). Given the state's large geographic area and distance to food resources particularly in rural areas, transportation challenges, such as vehicle access, pose a significant challenge to food access (32). The inclusion of transportation offers a deep understanding of the factors influencing socioeconomic disadvantage for senior populations in Central Texas. In addition, unlike Lee et al., who included the percentage of the population not receiving SNAP relative to those below the poverty line, the OASDI considers the percentage of older adults aged 60 and older receiving SNAP. Even though SNAP alleviates food insecurity amongst the older adult population, we are using this statistic as a measure of the potential risk of food insecurity in the region of interest. Texas has the second-highest food insecurity rate among the states, and there are stringent criteria for SNAP eligibility. Therefore, this study considered receipt of SNAP as an indication of unmet need for food among older adults.

Poverty has consistently been linked to food insecurity among older adults (45). New funding initiatives and research studies have highlighted persistent poverty as an important factor for health disparities and equity. This study is unique in mapping persistent poverty data to provide context for the index and assist in the further interpretation of the OASDI. An important point is that the distribution of the OASDI index follows persistent poverty patterns to some extent, but not always. For example, some neighborhoods in East Austin, which exhibit greater socioeconomic disadvantage, are also marked as experiencing persistent poverty. However, some tracts with lower index scores, indicating less socioeconomic disadvantage, are still marked as persistent poverty tracts. It is important to consider that OASDI is a snapshot in time, whereas the Persistent Poverty Indicator captures high poverty over time. The Persistent Poverty Indicator highlights areas facing prolonged economic challenges, while the OASDI provides a cross-sectional perspective for older adults and captures short-term demographic shifts that have been typical in Texas (Supplementary Figure S2). Additionally, this study determined the distribution of the OASDI at multiple levels for a 23-county region in Texas and is the first study of its kind to characterize socioeconomic disadvantage for older adults in this way. Results identified an outlier with extreme disadvantage located near areas with relatively less disadvantage. Previous studies have reported the difficulty in locating smaller “pockets” of disadvantage within a larger area (46, 47). Findings indicate the value of the index for identifying disparities within a large geographic area.

Regarding implications, this study is a critical part of the NUEVA project. Findings serve as a foundation for characterizing local capacity for senior nutrition within the 23-county study area and providing evidence needed to develop new policies, systems, and environmental interventions addressing food insecurity among older adults. Specifically, the new index will be used to evaluate a new app designed to connect senior meal providers with residents in Central Texas. Policymakers can apply findings to advocate for existing senior programs or the development of new programs to support older adults. The Older Americans Act funds critical services and supports enabling adults ≥60 years to live independently as they age. Stakeholders working with the local AAAs and COGs might make use of study findings for planning or design of new programs. Practitioners can integrate findings from this study to tailor strategies and solutions to the unique needs and assets of different communities. For example, service providers working with senior centers, health clinics and hospitals, community centers, and churches can incorporate findings to prioritize food security for older adults. While this study conducted member checking to obtain feedback about the validity of the index results, future directions include additional analyses to further validate the new index, as well as mixed methods research aimed at better understanding and addressing food insecurity for this population. We also plan to expand the timeline to study changes in the spatial pattern of the index over time. A longitudinal study will allow us to incorporate additional factors, such as living in multigenerational households, which has been shown to be associated with food insecurity among older adults (48).

In terms of limitations, two of the 12 index variables—unmet needs for food and housing—were proxy variables, meaning that the operational variables did not fully represent the conceptual variable due to data limitations. For example, the Census samples different counties to obtain state-level estimates of food insecurity as part of the Current Population Survey Food Security Supplement (CPS-FSS), but there are no comprehensive local data on food insecurity at the county or census tract levels (49). This study is unable to determine the extent that limited data influenced the findings. Findings must be interpreted carefully without speculating about the extent of housing or food insecurity among older adults. Future research might consider cost-effective ways to systematically collect data on food insecurity within a region, with the same measure used in the Census survey, the CPS-FSS. In addition, the housing variable was limited, but Census data on housing insecurity appears to be in progress. Based on a white paper in collaboration with the Department of Housing, the Census has pre-tested a housing insecurity module of the American Housing Survey (50) and updated a multi-dimensional hardship index, as an indicator of wellbeing for the adult population. The Multi-dimensional Hardship Index includes indicators of material hardship or unmet needs for employment, housing, mental health, and food (51). Researchers may benefit from these new questions and important data sources of housing insecurity going forward. Other variables used in the index, such as the variable for transportation, do not fully represent unmet needs for transportation among older adults, and additional work is needed to understand the importance of transportation networks for creating and distributing resources for mobility within a geographic region, as well as facilitating capabilities required for health (52). Lastly, the index creation used quantiles, which can exaggerate lows and highs. However, analyses showed that the distribution for variables in the index was normal, and the use of quantiles did not appear to impact the index. Lastly, there are likely important factors underlying the single outlier in Hays County, such as the distribution of households with adults ≥60 years or proximity to Interstate-35. Identifying and exploring those factors is beyond the scope of this manuscript, but additional investigation would be important for informing multi-level policy decisions.

## Conclusions

This study outlines the development of a replicable older adult socioeconomic disadvantage index (OASDI) for the 23 counties in Central Texas with a primary focus on identifying existing disparities within and across a large geographic region. Findings shows that there was more socioeconomic disadvantage among senior households in the San Antonio vs. the Austin metro area. In addition, the findings identified a small area of extreme socioeconomic disadvantage within Hays County. This study informs the ongoing development of an app to address food insecurity and social isolation among households with older adults and provides critical information to researchers, practitioners, and policymakers working to improve health and wellbeing of older adults within this region and state of Texas. This study also offers support for current Census efforts to develop a measure for housing insecurity and create a multidimensional hardship index as critical determinants of health. Given the existing disparities within and across planning regions, future work is required to prioritize and coordinate policy, systems, and environmental changes specifically for households with older adults.

## Data Availability

Publicly available datasets were analyzed in this study. This data can be found here: https://www.census.gov/. Table 1 in the article provides the census table number for the data used in this research.
