# Supplementary material for: A socioeconomic disadvantage index for informing policy, systems, and environmental change interventions for senior nutrition programs
Source: Front Public Health. 2025 Jan 21;13:1520925. doi: 10.3389/fpubh.2025.1520925 (PMC11790468; doi:10.3389/fpubh.2025.1520925)
Supplement: Supplementary file 1 [file Data_Sheet_1.docx]

SUPPLEMENTARY MATERIALS


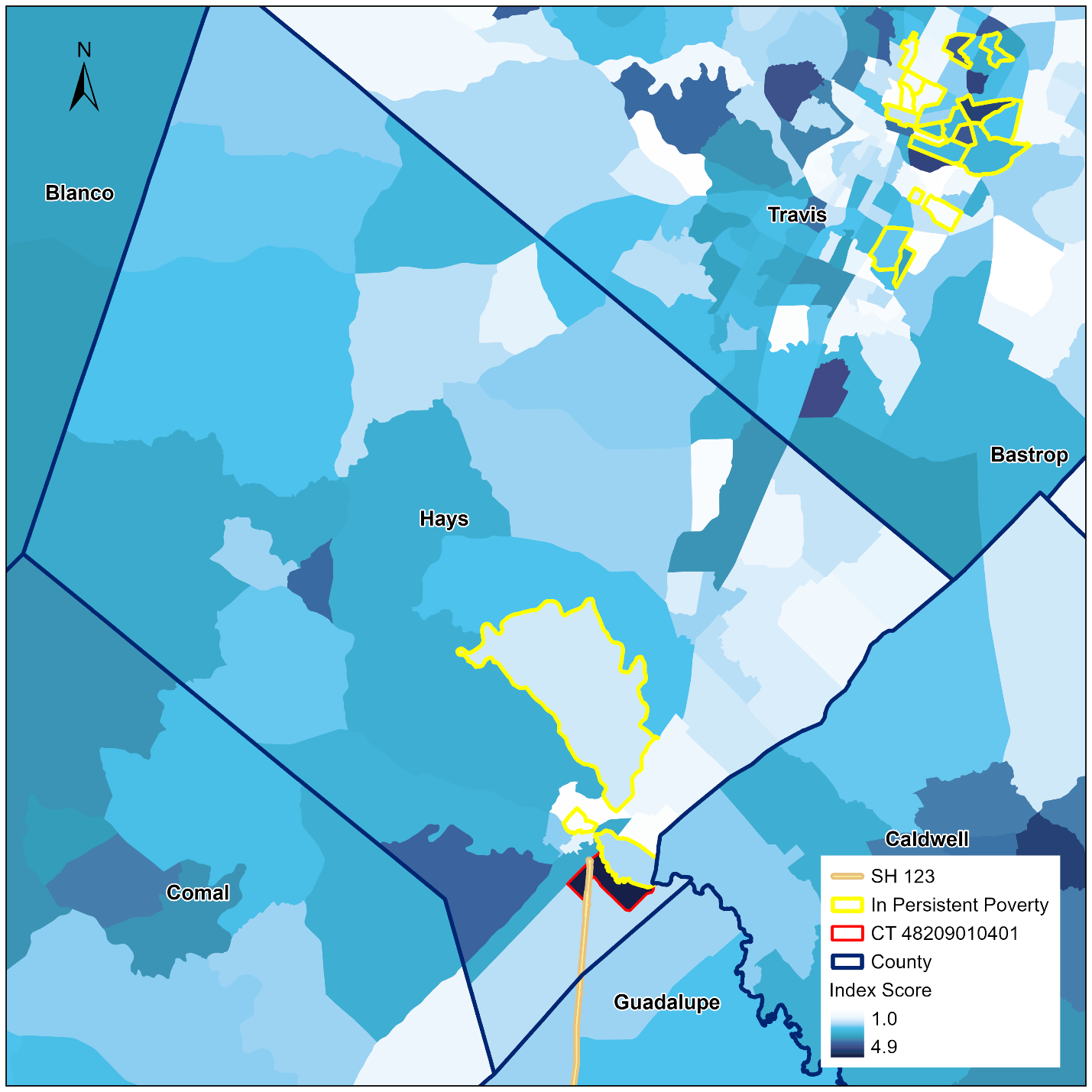


**Figure S1. Outlier from CAPCOG v. AACOG analysis.** The outlier analyses identified one outlier in Hays County in the area highlighted in fuchsia. The names of neighbouring counties – Blanco, Travis, Bastrop, Caldwell, Guadalupe, and Comal – -are shown on the map.


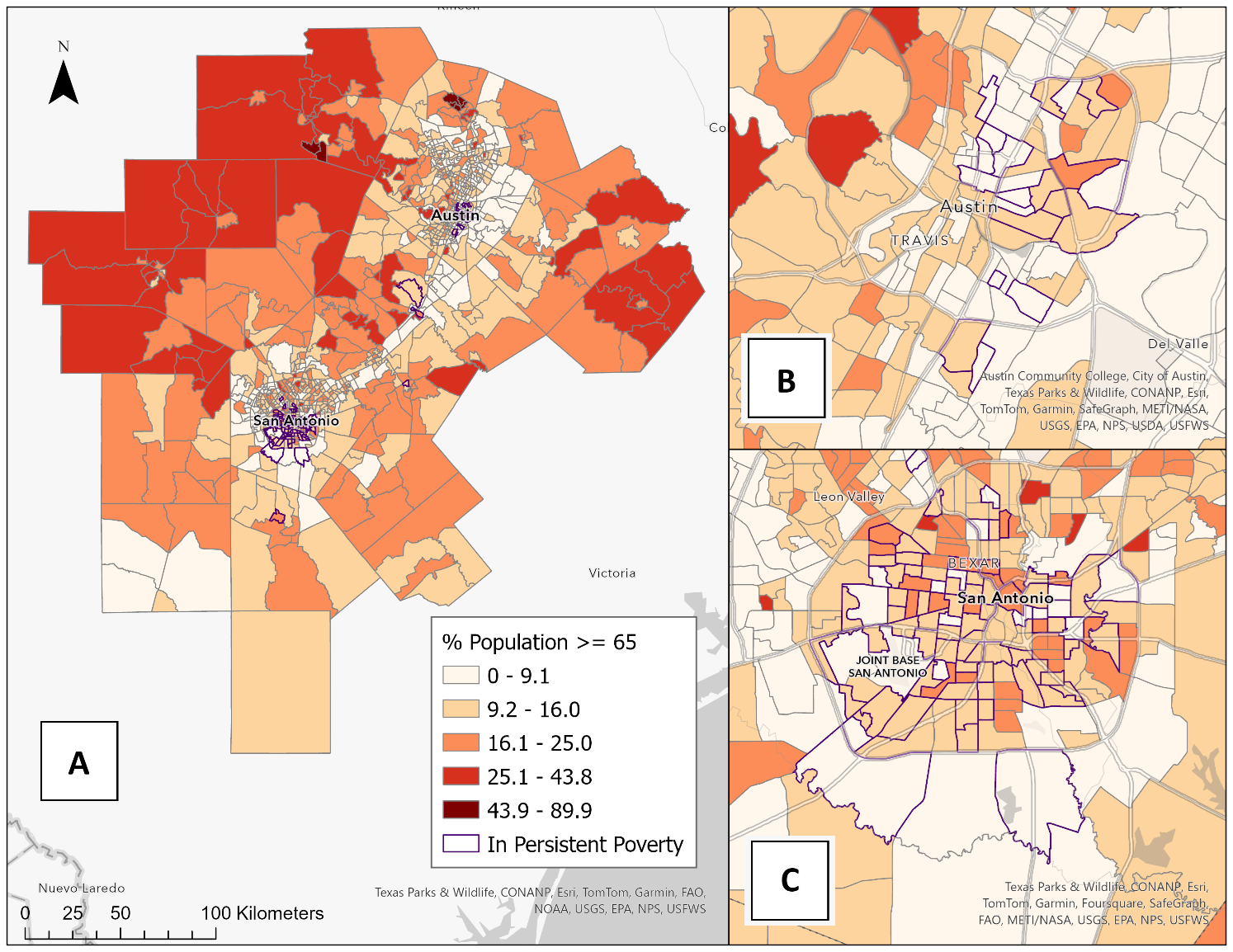
**Figure S2. Percentage of population aged 65 or older with persistent poverty**. Choropleth maps were utilized to show the population distribution of adults 65 or older within Central Texas. Darker colors indicate areas with lower percentage of population 65 and older while lighter colors show areas with greater percentages of older adults. The highlighted census tracts indicate persistent poverty. The highlighted census tracts indicate areas of persistent poverty. Section A of the map shows the population of 65 or older across the 23 counties in the study area. Section B shows a close-up view in Austin, Travis County. Section C shows a close-up view in San Antonio, Bexar County.
